# Meteorological Journal

**Published:** 1823-07

**Authors:** 


					METEOROLOGICAL JOURNAL,
From May 20, to June 19, 1823.
By Messrs. William Harris and Co. 50, Holborn, London.
Ma}
20'
21
22
23
24 O
25
26
27
28
29
30
31
June
1
2
3
4
5
6
7
3 ?
9
10
ll
12
13
14
15
16
17
18
19
Rain
gauge
.03
.02
.04
THERM.
29.49
29.39
29.50
29.57
29.68
29.46
29.50
29.74
29.82
29.92
29.97
30.01
29.95
29.71
29.41
29.26
29-31
29.62
29-90
29.73
29.74
29-76
29-87
29.74
29.66
29.70
29-80
30.06
30.10
30.03
29.94
29.44
29.50
29.61
29.71
29.62
29.48
29.61
.77
29.87
29.94
30.00
30.00
29.86
29.45
29.35
29.28
29.44
29.83
29-76
29.74
29.75
29.84
29.86
29.78
29.64
29.72
30.00
30.10
30.06
29.95
29.90
DE
LUC'S
HYG.
9 A.M
SSE
SVVva.
SSW v.
sw
sw
ssw
ssw
NNE
NE
ENE
SE
E
S
WSW
SW
sw
sw
w
sw
ssw
w
NW
NNE
NNE
N
N
N
N
N
N
NNE
10p.m.
SE
S var.
SW
W var.
SSW
ssw
NNW
N
NE
E
S
E
SSW
SW
ssw
sw
sw
NW
sw
WSW
w
NNE
NNE
E
NNE
NE
NE
ESE
NE
NNE
N
ATMOSPHERIC
VARIATION.
9 A.M.
Slio'ry
Fine
Slio'ry
Slio'ry
Cloud.
Cloud.
Fine
Fine
Fine
Fine
Fine
Fine
Fine
Fine
Fine
Fine
Fine
Cloud.
Fair
Fine
Fine
Fine
Fine
Fine
Fine
Fine
Cloud.
Fine
Fine
Cloud.
Cloud.
2 P.M.
Fine
Rain
Fine
Slio'ry
Slio'ry
Slio'ry
Fine
Cloud.
Cloud.
Fine
Cloud.
Fine
10p.m.
Slio'ry
Fine
Fine
Fine
Rain
Fail-
Fair
Rain
Fine
Cloud.
Fair
Cloud.
The quantity of rain during the month of May,
jwas 1 inch and 7.100ths.

				

